# Grasping like humans: a stacked-fabric grasp localization strategy based on convex structure perception and hierarchical fuzzy decision-making

**DOI:** 10.3389/frobt.2026.1917009

**Published:** 2026-10-05

**Authors:** Jinde Chen, Hubo Chu, Zhenyang Chao, Tie Zhang

**Affiliations:** 1 School of Mechanical and Automotive Engineering, South China University of Technology, Guangzhou, China; 2 Guangdong Zhensheng Technology Group Co., Ltd., Meizhou, China; 3 School of Mechanical Engineering, Guangdong Ocean University, Zhanjiang, China

**Keywords:** artificial intelligences, convex structure perception, fabric grasp localization, hierarchical fuzzy neural network, human behavior imitation

## Abstract

**Introduction:**

Due to the complex structures, variable morphologies, and multiple feasible grasping strategies for entangled fabrics during stacked sorting, achieving stable robotic grasping in such scenarios remains highly challenging. This study formulates the stacked-fabric grasping task as a problem of structural perception and multi-attribute decision-making.

**Methods:**

A stacked-fabric grasping method is proposed. Specifically, a convex-structure perception method based on point cloud geometric features is developed to extract operable regions from point cloud data, and a set of candidate grasp poses is generated based on these structures. Furthermore, to address the difficulty of effectively evaluating candidate poses, a hierarchical fuzzy neural network is developed by simulating the human fuzzy decision-making process, enabling interpretable evaluation and selection of the optimal grasp pose.

**Results:**

Comparative experiments are conducted on a constructed fabric grasping dataset. Compared with the best-performing baseline model, the proposed method improves the prediction accuracy of grasp feasibility, grasp separation effectiveness, and grasp reliability by 1.3%, 0.3%, and 0.7%, respectively. Robotic grasping experiments further show that the proposed method achieves a grasping success rate of 93.3% in randomly stacked scenarios.

**Discussion:**

The results demonstrate that integrating convex structure perception with hierarchical fuzzy decision-making provides an effective and interpretable strategy for grasp pose localization and selection in stacked-fabric grasping.

## 1 Introduction

In application scenarios such as domestic service, medical care, and industrial laundry, fabric sorting and handling are essential components of automation. However, fabrics are flexible, easily deformable, and prone to self-occlusion, which makes accurate grasp-pose localization in complex scenes highly challenging. As a key step in fabric sorting, grasp-pose estimation directly affects the stability and success rate of subsequent manipulation operations (Borràs, 2022; Sun, Huang, and Li, 2025; Gusseme et al., 2025).

Existing studies on grasp-pose generation can be broadly divided into two categories: end-to-end methods and staged methods (Longhini et al., 2025). End-to-end methods typically use deep learning models to directly predict grasp poses from perceptual inputs (Zhou et al., 2025a; Gu et al., 2026), or employ deep reinforcement learning to learn grasping strategies through interaction with the environment (Shehawy et al., 2023; Lee et al., 2024). Although these methods have achieved promising results in certain scenarios, their performance often depends on large-scale training data and costly interactions, and their decision processes usually lack interpretability. In contrast, staged methods decompose the grasping task into two subproblems: grasp-region perception and grasp-pose generation. This decomposition alleviates data dependence to some extent and improves interpretability by leveraging structural information and geometric constraints. Therefore, staged methods have been widely applied in fabric sorting tasks (Dželo et al., 2025; Wang H. et al., 2025).

In staged methods, accurate identification of grasp regions is a prerequisite for reliable grasp-pose generation. Existing studies usually localize grasp regions by extracting local fabric features, such as edges and corners, or semantic structural features, such as collars and cuffs (Han and He, 2024; Luo and Hu, 2026). However, in stacked and entangled fabric scenes, severe occlusion and morphological distortion often make it difficult to stably extract key regions with operational significance. In the grasp-pose selection stage, existing studies usually generate candidate grasp poses within the identified grasp regions based on fabric geometric information, and then select the optimal grasp pose using either rule-based evaluation functions or learning-based evaluation methods (Huang and Au, 2022). Manually designed rules have limited expressive capability and are difficult to use for characterizing the relationship between complex deformation and grasping outcomes. Learning-based methods, on the other hand, mostly rely on black-box models, lack explicit decision mechanisms, and therefore struggle to reflect the experience and preferences involved in human grasping.

By contrast, when humans handle complex fabric grasping tasks, they typically rely on local structural perception and experience-driven decision-making mechanisms. Specifically, humans first visually perceive raised structures on the fabric surface and localize key regions with operational significance in complex scenes. Then, by combining local geometric features with the overall stacking state, they comprehensively evaluate multiple candidate grasp poses and select the optimal grasp pose. This process demonstrates strong structural awareness and fuzzy decision-making characteristics. Inspired by this observation, this paper models stacked-fabric grasp-pose localization as two key processes, namely, structural perception and fuzzy decision-making, and proposes a grasp-pose localization method based on convex structure awareness and hierarchical fuzzy decision-making. The proposed method aims to improve the accuracy, robustness, and interpretability of grasp-pose localization in complex stacked-fabric scenes. The main contributions are as follows,

1. A staged grasp-pose localization framework for stacked-fabric sorting is designed, in which the complex fabric grasping problem is decomposed into two key processes: convex structure perception and candidate-pose selection. This framework enables robust grasp-pose localization in scenes with no fixed structural features and strong morphological uncertainty.
2. A convex structure perception method based on point cloud geometric features is proposed. By fusing curvature and normal-consistency information, the method stably extracts convex regions of fabrics, identifies structural regions with operational significance from complex entangled or stacked states, and constructs a candidate grasp-pose set satisfying structural constraints.
3. To address the problem of selecting the optimal grasp pose from a set of candidate poses, a hierarchical fuzzy neural network model is proposed by simulating the human fuzzy decision-making mechanism. The method fuses global scene information, local geometric features, and pose parameters to learn the membership distributions of multiple attributes, including graspability, stability, and separation capability. It then combines fuzzy inference to achieve multi-attribute fusion, thereby enabling interpretable evaluation and selection of the optimal grasp pose.

The remainder of this paper is organized as follows. Section 2 reviews related work, focusing on fabric grasp-region perception methods and grasp-pose evaluation and selection methods, and analyzes the limitations of existing methods in complex entangled fabric scenes. Section 3 presents the overall framework of the proposed method, detailing the point cloud geometric feature-based convex structure perception method, the candidate grasp-pose generation process, and the grasp-pose selection strategy based on the hierarchical fuzzy neural network. Section 4 introduces the dataset construction and experimental setup, presents the model training details, and verifies the performance advantages of the proposed method in terms of optimal grasp-pose localization and multi-attribute evaluation through comparative and ablation experiments. Section 5 concludes the paper.

## 2 Related work

### 2.1 Grasping region perception

In fabric sorting tasks, accurate identification of grasp regions is a prerequisite for stable manipulation. This is particularly true in stacked and entangled fabric scenes, where severe occlusion, self-occlusion, and morphological distortion cause fabric surfaces to exhibit highly complex and irregular structural characteristics. As a result, extracting grasp regions with operational significance from raw perceptual data becomes a key challenge.

Existing studies have developed various grasp-region perception methods based on local fabric features, semantic structures, and geometric structures. One line of research extracts potential grasp regions by identifying local features on garment surfaces, such as edges, wrinkles, and corners (Corona et al., 2018). For example, Qian et al. (2020) transformed the conventional edge detection problem into a pixel-level semantic segmentation problem, enabling the identification of true garment edges and corner regions through category prediction for each pixel. Shehawy et al. (2023) proposed a visual perception method based on corner detection to identify key grasping points on towels, thereby localizing grasp regions. Tabernik et al. (2024) proposed a grasp-point detection method based on center-direction regression, which learns the direction field from image pixels to target points to achieve corner-level grasp-point detection. However, such methods mainly rely on local features or heuristic geometric cues and lack explicit modeling of grasp stability and operability, making them prone to misjudgment in complex deformation or multilayer stacking scenarios.

Another line of research focuses on the structural semantic information of garments. By segmenting key structures, such as collars and cuffs, these methods constrain the grasping space, thereby improving manipulation stability and task adaptability. Ramisa et al. (2016) constructed a bag-of-visual-words representation using image appearance features and combined it with a classifier to detect collars. Ren et al. (2023) proposed a grasp-oriented fine-grained garment segmentation method, in which U-Net was used to perform pixel-level classification on depth maps, enabling the segmentation of key garment edge regions such as necklines, cuffs, and hems. Li et al. (2025) proposed a YOLO11-based collar recognition method to determine grasp regions. However, in stacked and entangled scenes, severe occlusion, self-occlusion, and morphological distortion often make key structures difficult to accurately identify from visual information, thereby limiting the applicability of such methods.

From the perspective of general-purpose fabric grasping, identifying geometric structural features with cross-category generalization capability has become a key issue for achieving reliable grasp-region perception in stacked and occluded scenes. To improve generalization in complex scenes, some studies have begun to focus on the geometric structural features of fabrics. Caporali and Palli (2018) proposed a point cloud-based grasp-pose identification method, in which an entropy map was constructed to characterize wrinkle distributions on the fabric surface. Graspable regions were then extracted by combining convexity and depth information, and grasp poses were generated by fitting curves to the wrinkles. Zhu et al. (2025) proposed a garment grasping framework for dark environments, in which garment regions were perceived through infrared semantic segmentation. Combined with geometric wrinkle analysis of depth maps, the most prominent position was selected from candidate regions as the grasping point. Compared with local feature-based and semantic structure-based methods, geometric structural features such as protrusions and wrinkles exhibit stronger cross-category generalization capability, providing an important basis for effective grasp-region identification in stacked fabrics.

However, existing methods are mostly based on heuristic geometric features or local structural metrics and mainly focus on structure detection or saliency evaluation. They lack unified modeling of the relationship between structures and grasping behavior, making it difficult to stably characterize truly operable structural regions under complex stacking and entanglement conditions. Therefore, how to extract structural features with explicit operational constraints from point cloud data and construct candidate grasp regions closely associated with grasping behavior remains a key problem in current research.

### 2.2 Grasping pose generation

After the grasp region is determined, the generation and selection of grasp poses directly affect the stability of the grasping process and the final manipulation outcome. Because fabrics exhibit complex non-rigid deformation, multiple potentially feasible grasp poses may exist even within the same local region. Therefore, selecting the optimal solution from the candidate poses becomes a key issue.

Existing studies have mainly investigated grasp-pose selection using geometric rules, manually designed evaluation functions, and learning-driven value functions. Geometric rule-based methods usually construct grasp poses according to local geometric and physical information. Chen et al. (2023) identified the collar region and determined the collar center region through skeleton extraction. Within this region, the point with the most prominent wrinkle was selected as the grasping position based on local point cloud curvature. Local PCA analysis was then used to obtain the normal and principal directions, thereby constructing the grasp pose. Li et al. (2026) obtained the three-dimensional position of the fabric through visual detection and binocular depth reconstruction, and determined the grasping position using either the region center or the local highest point. Tactile feedback was then incorporated to adjust the grasp pose online during execution. Such methods construct grasp poses based on geometric and physical information and thus offer good interpretability; however, their expressive capability is limited in complex deformation scenarios.

To improve the effectiveness of candidate-pose selection, some studies have introduced manually designed evaluation functions to score candidate poses. Zhu et al. (2025) modeled wrinkle structures on the fabric surface and searched for points satisfying protrusion characteristics within candidate regions. A protrusion metric function based on local height differences was then constructed to score candidate poses, and the position with the highest score was selected as the grasp pose. Ngo et al. (2026) proposed a fabric manipulation method based on finite element analysis and sequential optimization from the perspective of global deformation control. By constructing a flatness evaluation metric and optimizing multiple grasping points and their execution sequence in simulation, the method achieved flat unfolding of the fabric. Huang and Au (2022) proposed a task-driven grasp-position evaluation metric. By constructing a local action function based on distance and direction and integrating the grasping effect along the deformation path, a grasp-position quality index was defined to select the optimal grasping position from candidate regions. However, such methods rely on manually designed evaluation functions with limited expressive capability, making it difficult to accurately characterize the relationship between fabric deformation and grasping effects in complex scenes.

To address these limitations, researchers have further introduced learning-driven methods for grasp-pose selection. Zhou et al. (2025b) introduced a reinforcement learning method to obtain reward signals through interaction with the environment and learn a pixel-level action-value function. The expected return of performing actions at different positions was then evaluated, thereby enabling adaptive selection of grasp poses. Huang, Efendi, and Wang (2025) formulated the grasp-pose selection problem as a pixel-level action-value estimation problem. A deep Q-network was used to predict the expected return of performing grasping at each position, and the optimal grasp pose was selected by maximizing the learned Q-value. Choi et al. (2025) modeled grasp-pose selection as an optimization problem constrained by material properties. By predicting post-grasp deformation and folding risk, the optimal grasping point was searched for within the constrained space. Compared with rule-based and manually designed evaluation methods, learning-based methods have stronger expressive capability; however, they usually rely on large amounts of data. In particular, reinforcement learning algorithms remain highly data-dependent, and their training stability still requires further improvement.

The above methods mostly rely on black-box models or manually designed rules for grasp-pose selection, making it difficult to simultaneously achieve strong complex-feature modeling capability and interpretability in the decision process. In recent years, some studies have introduced fuzzy theory into robotic grasping to enhance the representation of uncertain information and the transparency of decision-making. Kuo and Chen (2023) used Gaussian and Sigmoid membership functions to fuzz continuous observations such as the robot hand, object, and target position, and combined them with reinforcement learning algorithms to learn grasping and placement strategies. Wang S. et al. (2025) proposed a deep spiking Takagi-Sugeno-Kang fuzzy network, which extracts multimodal temporal features through a deep spiking network and identifies grasping states such as shaking, stability, and slippage using learnable T fuzzy rules. However, the above fuzzy rules mainly focus on input encoding or high-dimensional hidden feature extraction, and are difficult to establish intuitive correspondence with specific grasp attributes. Wei et al. (2026) further constructed a state-action fuzzy controller that generates hierarchical grasp-force adjustment commands according to the current and historical grasping states, achieving a mapping from semantic states to interpretable control actions. However, this method mainly targets force adjustment during the grasp execution stage, with a relatively single decision objective and a lack of comprehensive trade-offs among multiple grasp attributes. Therefore, how to extract and fuse effective features from multi-source heterogeneous information and realize multi-attribute evaluation of candidate grasp poses remains a key problem to be solved in complex stacked-fabric grasping.

## 3 Proposed methods

### 3.1 Framework overview

To address the difficulty of pose selection caused by structural complexity and multiple feasible grasp poses in stacked and entangled fabric grasping, this paper proposes a grasp-pose localization method based on convex structure awareness and hierarchical fuzzy decision-making. The overall framework is shown in Figure 1. The proposed method adopts a staged modeling strategy and mainly consists of two key stages. First, structural perception is performed on the fabric surface based on point cloud geometric features to extract convex structural regions with operational significance, from which a set of candidate grasp poses satisfying structural constraints is generated. Second, a hierarchical fuzzy neural network is constructed to evaluate the candidate poses. By fusing global scene information, local geometric features, and pose parameters, the model learns multi-attribute membership distributions and selects the optimal grasp pose through fuzzy inference. Through this staged modeling strategy, the unstructured and highly uncertain grasping problem is transformed into a multi-attribute decision-making problem under structural constraints, thereby improving the robustness and interpretability of grasp-pose selection while ensuring the physical feasibility of candidate poses.

**FIGURE 1:**
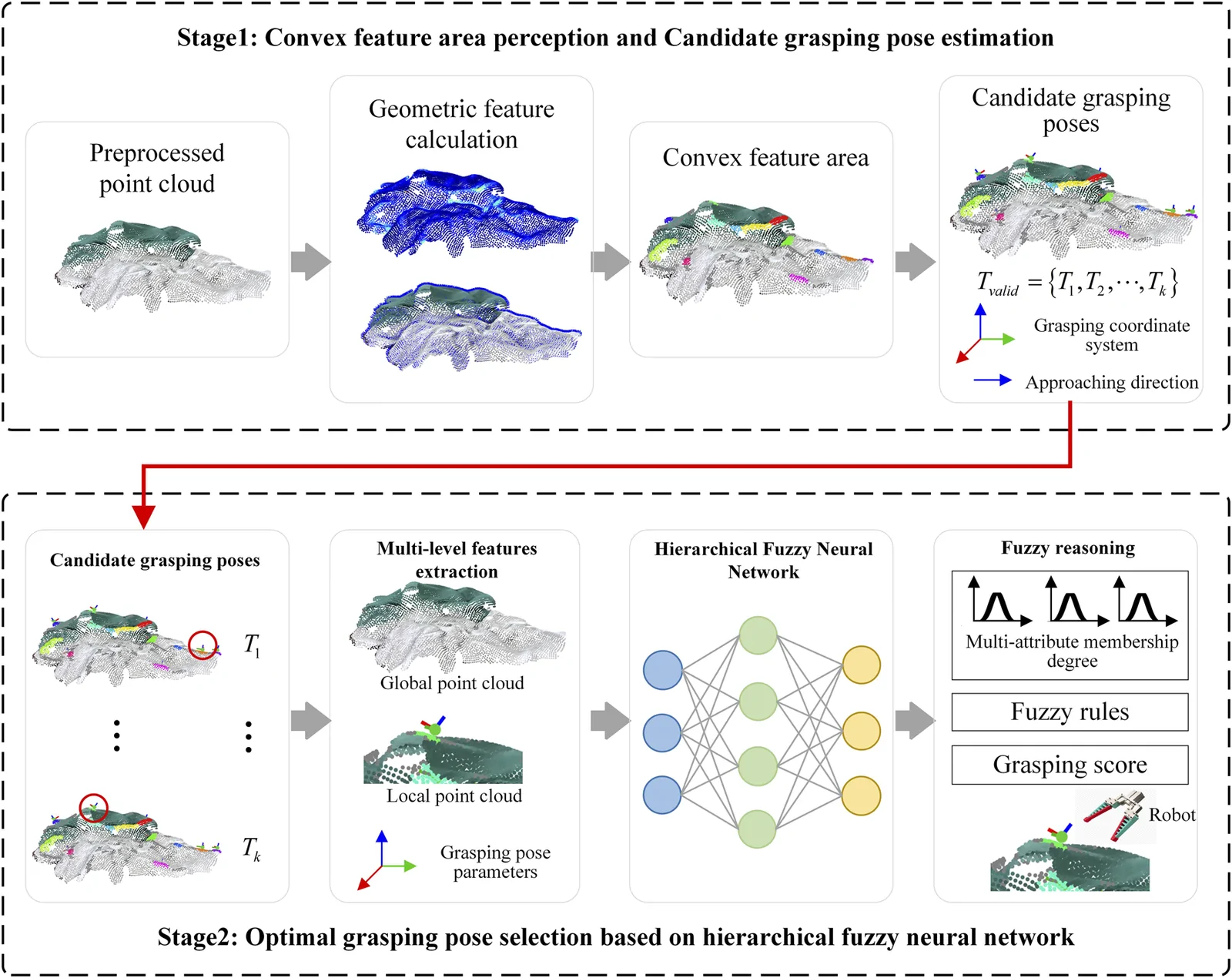
Framework Overview. In the first stage, convex feature regions are extracted from the preprocessed point cloud and candidate grasp poses are generated. In the second stage, the global point cloud, local point cloud, and pose parameters are fused. Multi-attribute membership degrees are estimated through the hierarchical fuzzy neural network, and the optimal grasp pose is selected through fuzzy inference.

### 3.2 Fabric convex-region perception and candidate-pose generation based on point cloud geometric features

#### 3.2.1 Perception of fabric convex feature regions

In entangled fabric scenes, determining the grasp pose first depends on effective perception of local operable structures. Because fabric surfaces contain numerous wrinkles, stacks, and occlusions, it is difficult to directly locate regions with grasping significance using only the raw point cloud. Therefore, starting from local geometric structures, this paper proposes a fabric convex feature-region perception method to extract convex connected regions with operational value from complex fabric surfaces and provide structural constraints for subsequent candidate grasp-pose generation. The perception process consists of three steps, as shown in Figure 2.

**FIGURE 2:**
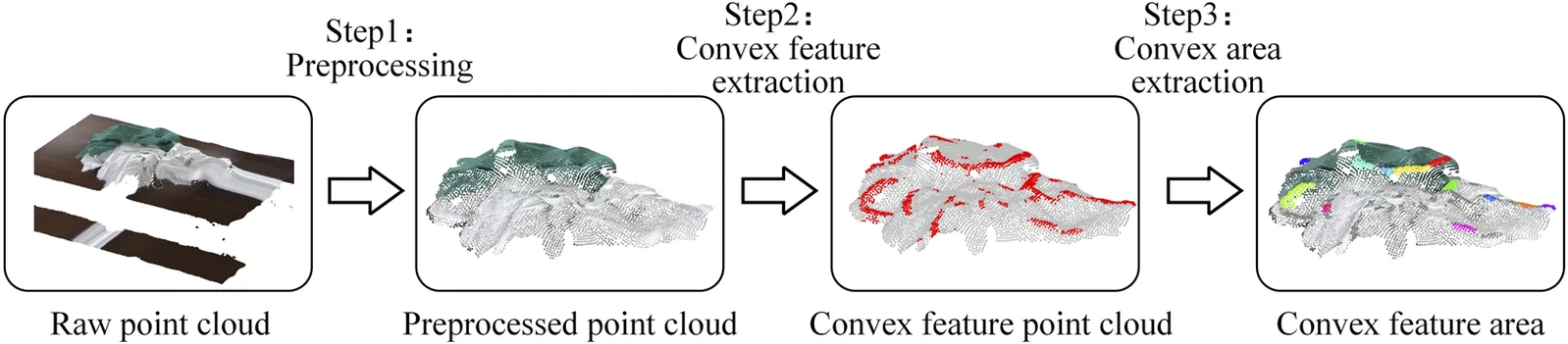
Extraction Process of Fabric Convex Feature Regions. The raw fabric point cloud is taken as input and processed sequentially through point cloud preprocessing, convex feature point extraction, and convex feature region clustering, yielding the convex feature regions on the fabric surface as output.

##### 3.2.1.1 Point cloud preprocessing

Voxel downsampling and statistical filtering are used to preprocess the raw point cloud, removing redundant points and outlier noise and obtaining the target point cloud within the workspace. The preprocessed fabric point cloud can be expressed as shown in Equation 1.

$$P={\left\{p_{i}\right\}}_{i=1}^{N}$$ (1)

where P denotes the preprocessed fabric point cloud set in the current scene, $p_{i}$ denotes the i -th point, and N denotes the total number of points in the point cloud.

##### 3.2.1.2 Convex feature point cloud extraction

Feasible grasping points on fabrics are usually associated with high-curvature feature points. To extract high-curvature points, a KD-tree is used to search for the neighboring points of each point $p_{i}$ , thereby constructing a local region $N\left(p_{i}\right)$ . In this paper, the number of neighboring points is set to 30. Principal component analysis (PCA) is then used to model the geometric distribution of the local neighborhood, and the eigenvalues and eigenvectors of the covariance matrix are obtained to characterize the geometric variations of the local surface in the normal and tangent-plane directions. For approximately flat regions, the point cloud dispersion in the normal direction is very small, and the corresponding eigenvalue is usually close to zero, whereas the eigenvalues in the two orthogonal directions of the tangent plane are relatively large. For locally curved regions, the dispersion in the normal direction increases, and the corresponding eigenvalue increases accordingly. Based on this property, the local point cloud curvature is defined in Equation 2.

$$H_{i}=\frac{\lambda_{i}^{n}}{\lambda_{i}^{n}+\lambda_{i}^{t1}+\lambda_{i}^{t2}}$$ (2)

Where $H_{i}$ denotes the curvature of the local region, $\lambda_{i}^{n}$ , $\lambda_{i}^{t1}$ and $\lambda_{i}^{t2}$ denote the eigenvalues corresponding to the normal direction and the two orthogonal directions of the tangent plane, respectively. The eigenvalues are obtained using PCA. In general, a larger curvature indicates more pronounced local bending deformation and a higher probability that the region contains wrinkles or protrusions. A curvature threshold $H_{th}$ is further introduced, and local point cloud regions with curvature greater than the threshold $H_{th}$ are extracted to construct the high-curvature point cloud set $P_{h}=\left\{p_{i}|H_{i}>H_{th}\right\}$ .

High-curvature regions may contain both concave and convex variations, whereas convex feature points are more suitable for grasping. To further extract convex feature points from the high-curvature point cloud set, a point cloud convexity criterion is proposed, whose mathematical expression is given as shown in Equation 3.

$$C\left(p_{i}\right)=n_{i}\cdot \left(p_{i}-p_{mean}\right)$$ (3)

where $n_{i}$ denotes the normal direction of point $p_{i}$ , and $p_{mean}$ denotes the geometric center of the local region $N\left(p_{i}\right)$ . When the discriminant C<0 , the current point is located in a locally convex region, otherwise, it is located in a locally concave region. Based on the above process, an effective convex feature point set can be extracted from the high-curvature point cloud set and expressed as shown in Equation 4.

$$P_{c}=\left\{p_{i}|H_{i}>H_{th},C\left(p_{i}\right)<0\right\}$$ (4)

##### 3.2.1.3 Convex feature-region extraction

Since the extracted convex feature points are still discretely distributed in space, they cannot be directly used to generate candidate grasp poses. To further obtain convex feature regions from the convex feature point set, convex feature-region extraction is modeled as an unsupervised clustering problem based on joint geometric features. This modeling is based on the observation that points on convex ridges of the fabric surface are spatially adjacent and have similar normal directions, principal tangent directions, and curvature distributions. For a convex feature point $p_{i}\in P_{c}$ , its joint geometric feature is constructed using Equation 5.

$$f_{i}=\left\{x_{i},n_{i},t_{i},H_{i}\right\}$$ (5)

where $x_{i}$ denotes the spatial coordinates of the point $p_{i}$ , $n_{i}$ denotes the normal direction, $t_{i}$ denotes the local principal tangent direction, and $H_{i}$ denotes the curvature. Because the principal tangent direction is extracted using PCA, its orientation has sign ambiguity; that is, two opposite directions are geometrically equivalent. Therefore, a sign-alignment function is introduced to unify the orientation of the principal tangent direction. Subsequently, the spatial coordinates and curvature values are scale-normalized.

To achieve autonomous perception of convex feature regions, the Density-Based Spatial Clustering of Applications with Noise (DBSCAN) algorithm is used to cluster the convex feature point set in the joint geometric feature space. For any two points, their distance in the feature space is defined in Equation 6 as,

$$d\left(p_{i},p_{j}\right)={\left\|f_{i}-f_{j}\right\|}_{2}$$ (6)

where $f_{i},f_{j}$ denote the joint geometric features of any two convex feature points, which are calculated using Equation 5. Given a neighborhood radius ε and a minimum number of samples MinPts , if the number of samples within the neighborhood of point satisfies $N_{\varepsilon }\left(p_{i}\right)\geq MinPts$ , then $p_{i}$ is defined as a core point. If point $p_{j}$ lies within the neighborhood of the core point $p_{i}$ , then $p_{j}$ is said to be directly density-reachable from $p_{i}$ . When there exists a point chain composed of several core points such that adjacent points on the chain satisfy the direct density-reachability relationship, these points are considered to belong to the same cluster region. Based on this principle, all density-reachable points are recursively searched and assigned to the same cluster, thereby partitioning the convex feature point set into several geometrically consistent convex feature regions $A_{k}$ , as denoted in Equation 7.

$$A=\left\{A_{1},A_{2},\cdot \cdot \cdot ,A_{k}\right\}$$ (7)

In summary, by constructing a joint geometric feature representation and combining it with a density-based unsupervised clustering mechanism, this paper enables the automatic organization of discrete convex feature points into region-level convex structural units.

#### 3.2.2 Candidate grasp pose generation

Fabric convex feature regions not only reflect local geometric undulation characteristics, but also implicitly contain directional information of operable structures. Therefore, estimating fabric grasp poses based on convex feature regions is an effective approach. For each convex feature region $A_{k}$ , its corresponding grasp pose can be expressed as shown in Equation 8.

$$T_{k}=\left(R_{k},o_{k}\right)\in SE\left(3\right)$$ (8)

Where $o_{k}$ is defined as the actual grasp center position, and $R_{k}$ denotes the rotation matrix of the grasp pose. To ensure that the gripper can approach the target region from outside the object during grasping, $o_{k}$ is obtained by summing the geometric center of all points in the convex feature region and the offset along the approach direction, as expressed in Equation 9.

$$o_{k}={\hat{o}}_{k}+da_{k}{z_{k}}^{\prime }$$ (9)

where ${\hat{o}}_{k}$ denotes the geometric center of the convex feature region; $da_{k}$ denotes the grasp offset; ${z_{k}}^{\prime }$ denotes the approach direction. ${\hat{o}}_{k}$ is calculated using Equation 10.

$${\hat{o}}_{k}=\frac{1}{N_{A_{k}}}\sum_{p_{i}\in A_{k}}p_{i}$$ (10)

Where $N_{A_{k}}$ denotes the number of points in the regional point cloud; $p_{i}={\left[x_{i},y_{i},z_{i}\right]}^{T}$ denotes a point in the point cloud within the convex region $A_{k}$ .

$da_{k}$ is defined as the projection distance between the highest point and the geometric center of the fabric convex region $A_{k}$ along the approach direction, minus the gripper length l , its mathematical expression is given in Equation 11 as,

$$da_{k}=\left[\underset{p_{i}\in A_{k}}{\max\left(p_{i}\cdot {z_{k}}^{\prime }\right)}-\left({\hat{o}}_{k}\cdot {z_{k}}^{\prime }\right)\right]-l$$ (11)

where l denotes the gripper length. Through Equation 11, the grasp offset can be adaptively adjusted, thereby reducing the risk of collision between the gripper and the fabric during the approach process and ensuring effective contact between the gripper and the target fabric. A schematic diagram of the grasp offset is shown in Figure 3.

**FIGURE 3:**
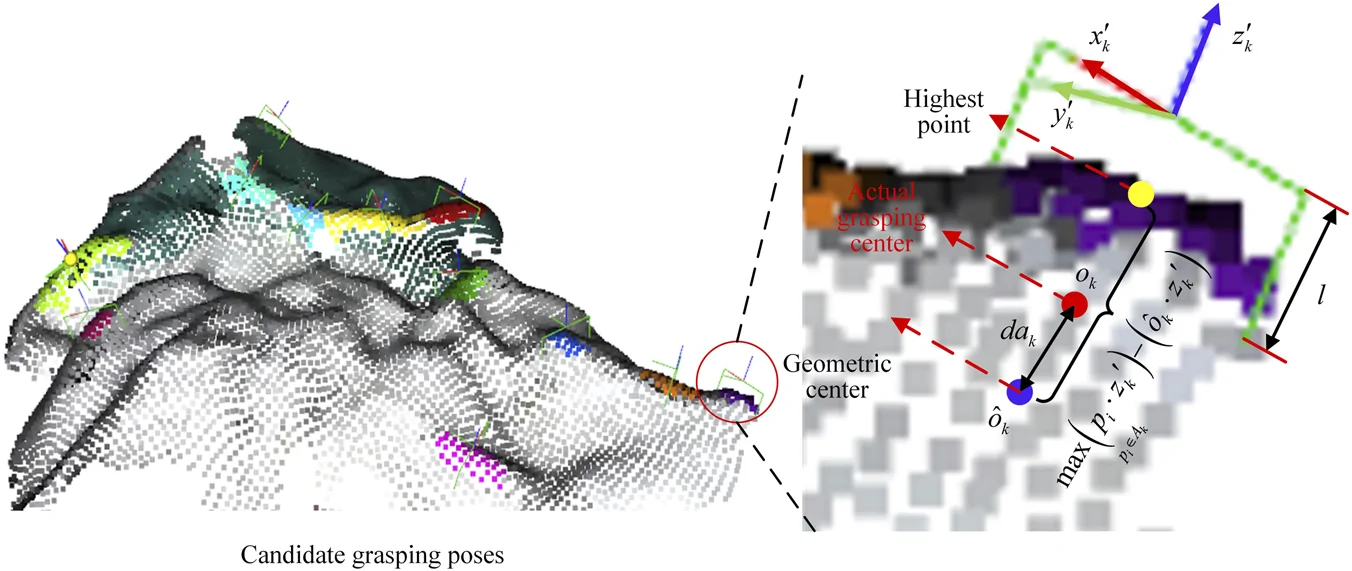
Visualization of candidate grasp poses. Regions in different colors represent the extracted convex feature regions, and the coordinate frames correspond to the candidate grasp poses of the respective convex feature.

The rotation matrix $R_{k}$ of the grasp pose is defined using the local geometric directions within the region, as expressed in Equation 12.

$$\begin{array}{l} R_{k}=\left[x_{k}^{\prime },y_{k}^{\prime },z_{k}^{\prime }\right]\\ \left\{\begin{array}{l} z_{k}^{\prime }=\frac{1}{\left|A_{k}\right|}\sum_{p_{i}\in A_{K}}n_{i}\\ y_{k}^{\prime }=\frac{1}{\left|A_{k}\right|}\sum_{p_{i}\in A_{K}}t_{i}\\ x_{k}^{\prime }=\frac{y_{k}^{\prime }\times z_{k}^{\prime }}{\left\|y_{k}^{\prime }\times z_{k}^{\prime }\right\|} \end{array}\right. \end{array}$$ (12)

where $z_{k}^{\prime }$ denotes the grasp approach direction, $y_{k}^{\prime }$ denotes the ridge extension direction, and $x_{k}^{\prime }$ denotes the gripper opening direction.

Furthermore, to ensure the feasibility of the generated grasp poses for practical implementation, a grasp-width constraint is introduced to filter the generated poses. Specifically, all points within the convex feature region $A_{k}$ are projected onto the gripper opening direction $x_{k}^{\prime }$ . If the projected width of the region along the gripper opening direction exceeds the maximum gripper opening width $w_{a}$ , the gripper cannot enclose the region from both sides. Therefore, grasp poses that do not satisfy the grasp-width constraint are removed, resulting in an operationally feasible candidate pose set $T_{valid}=\left\{T_{1},T_{2},\cdot \cdot \cdot ,T_{k}\right\}$ . The estimated candidate poses are shown in Figure 3.

### 3.3 Optimal grasping pose selection based on a hierarchical fuzzy neural network

Because fabrics are flexible, easily deformable, and lack fixed structural patterns, different candidate poses exhibit significant differences in grasp feasibility, grasp stability, and single-layer separation capability. Evaluation methods that rely solely on geometric features cannot comprehensively characterize the quality of candidate poses. Humans can effectively select an appropriate grasp pose in complex scenes based on multidimensional perceptual information and experiential knowledge. Inspired by the human decision-making mechanism, this paper models grasp-pose selection as a hierarchical multi-attribute decision-making problem and proposes an optimal grasp-pose selection method based on a hierarchical fuzzy neural network.

#### 3.3.1 Modeling of the human decision-making strategy

In the fabric grasp-pose evaluation task, expert decision-making relies on both local geometric details and global scene structure for comprehensive judgment. For each candidate grasp pose, experts first form a preliminary fuzzy judgment of grasp feasibility based on the local geometric morphology and contact characteristics of its neighborhood. They then combine the overall stacking state represented by the global point cloud with neighborhood distribution patterns and potential interference risks to obtain a fuzzy assessment of global constraints. On this basis, key attributes, including grasp feasibility, grasp stability, and single-layer separation capability, are represented by fuzzy membership degrees and integrated through comprehensive reasoning. Based on fuzzy linguistic variables such as High, Medium, and Low, the overall quality of each candidate pose is evaluated and ranked.

Based on the above analysis, the expert decision-making process for fabric grasp poses can be modeled as a fuzzy attribute mapping and reasoning process that fuses multi-level perceptual information, as shown in Figure 4.

**FIGURE 4:**
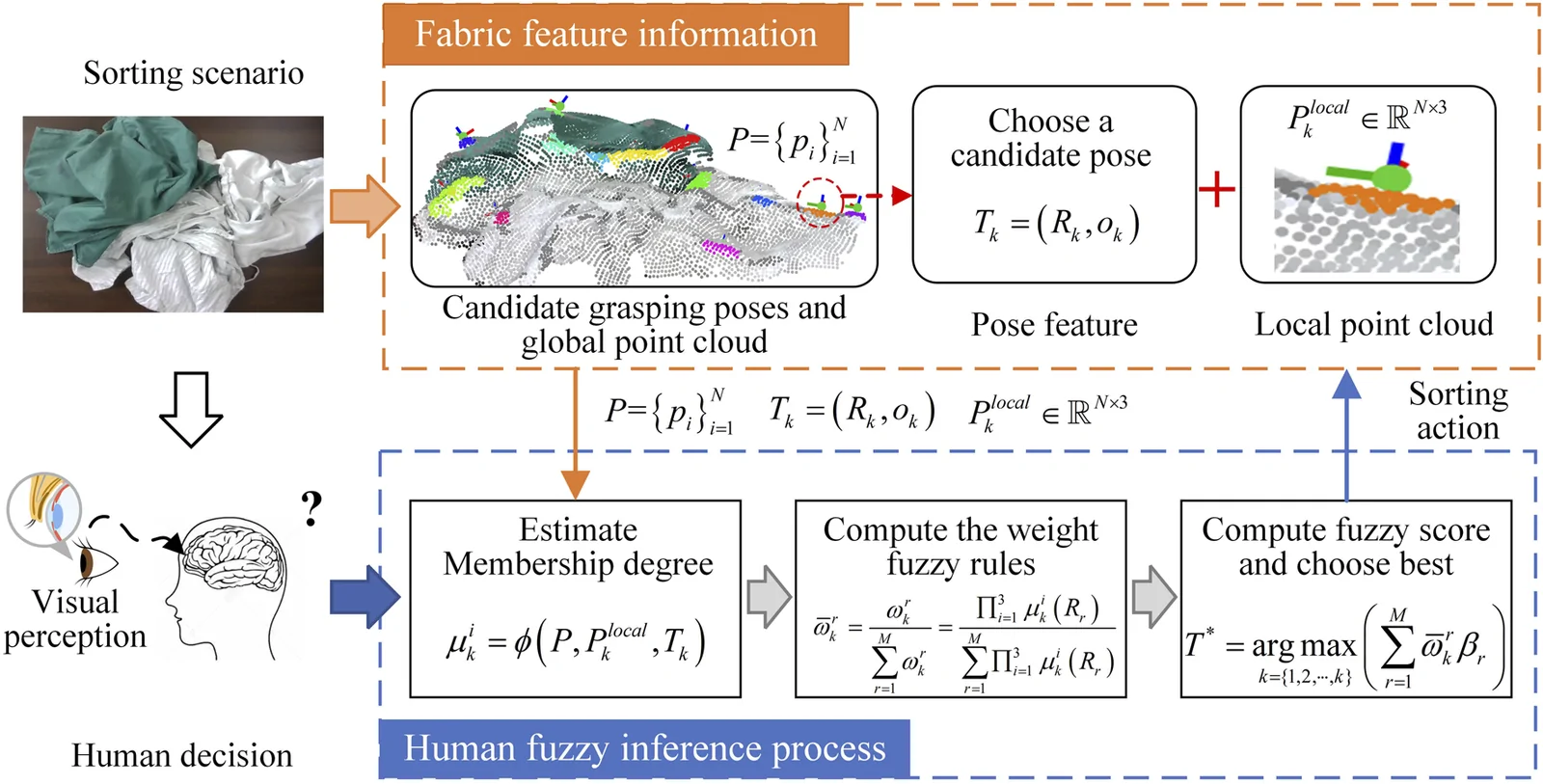
Expert Decision-Making Process for Fabric Grasp Poses. The orange dashed box represents fabric feature information, including the global point cloud, candidate grasp poses, and their corresponding local point clouds. The blue dashed box represents the human fuzzy inference process, including attribute membership estimation, fuzzy-rule weight calculation, and comprehensive scoring together with optimal grasp-pose selection.

Specifically, for each candidate pose $T_{k}=\left(R_{k},o_{k}\right)$ , its grasp center is used as the reference point, and a local point cloud region $P_{k}^{local}\in R^{N\times 3}$ is extracted within its neighborhood using the KD-tree method, where N denotes the number of points in the local region. Subsequently, the preprocessed global point cloud $P={\left\{p_{i}\right\}}_{i=1}^{N}$ is obtained using the method described in Section 3.1. On this basis, each candidate pose is described from three dimensions, namely, grasp feasibility, grasp stability, and grasp separation potential, and the attribute set is defined in Equation 13 as

$$F_{k}=\left\{F_{k}^{1},F_{k}^{2},F_{k}^{3}\right\}$$ (13)

where $F_{k}^{1},F_{k}^{2},F_{k}^{3}$ correspond to the three evaluation attributes, respectively. Since the human evaluation process is fuzzy, each attribute is represented by a fuzzy variable, i.e., $F_{k}^{i}=\left\{Low,Medium,High\right\}$ . Furthermore, the global point cloud image I , the candidate pose $T_{k}$ , and the corresponding local point cloud $P_{k}^{local}$ are used as inputs to learn the membership degrees of each attribute for the grasp pose. The mathematical formulation is expressed in Equation 14 as,

$$\mu_{k}^{i}=\phi \left(I,P_{k}^{local},T_{k}\right),i=\left\{1,2,3\right\}$$ (14)

where $\mu_{k}^{i}$ denotes the membership degree corresponding to the i -th attribute, and $\phi \left(\cdot \right)$ denotes the membership mapping function. To achieve joint reasoning over multi-attribute information, experts construct a fuzzy rule set $R={\left\{R_{r}\right\}}_{r=1}^{M}$ based on expert experience. Each rule corresponds to a linguistic combination of the three attributes, namely, grasp feasibility, grasp stability, and grasp separation potential, over {Low, Medium, High}. For a candidate pose $T_{k}$ , its activation strength under rule $R_{r}$ is calculated as the product of the membership degrees of the three attributes. The corresponding rule weights are then normalized according to Equation 15.

$${\bar{\omega }}_{k}^{r}=\frac{\omega_{k}^{r}}{\sum_{r=1}^{M}\omega_{k}^{r}}=\frac{\prod_{i=1}^{3}\mu_{k}^{i}\left(R_{r}\right)}{\sum_{r=1}^{M}\prod_{i=1}^{3}\mu_{k}^{i}\left(R_{r}\right)}$$ (15)

Finally, based on the expert rule score $\beta_{r}\in \left[0,1\right]$ of each rule $R_{r}$ , the score of each candidate pose is quantified. The score of a candidate pose is calculated as the weighted sum of all rule scores. Scores are computed for all candidate poses, and the candidate pose with the highest score is selected as the optimal grasp pose, as expressed in Equation 16.

$$T^{*}=\underset{k=\left\{1,2,\cdot \cdot \cdot ,k\right\}}{\mathrm{argmax}}\left(\sum_{r=1}^{M}{\bar{\omega }}_{k}^{r}\beta_{r}\right)$$ (16)

#### 3.3.2 Design of the hierarchical fuzzy neural network

Because fabrics are flexible, easily deformable, and lack fixed structural patterns, candidate grasp poses are difficult to evaluate using precise thresholds for attributes such as grasp feasibility, grasp stability, and single-layer separation capability. Therefore, the attribute evaluation process exhibits significant fuzziness and nonlinearity. To avoid the subjectivity and limited adaptability introduced by manually designed membership functions, this paper proposes a Hierarchical Fuzzy Neural Network (HFNN). The HFNN automatically learns attribute membership distributions at three levels, namely, global scene structure, local geometric morphology, and candidate-pose parameters, and further integrates fuzzy rule-based reasoning to obtain comprehensive scores for candidate poses. Its overall structure is shown in Figure 5.

**FIGURE 5:**
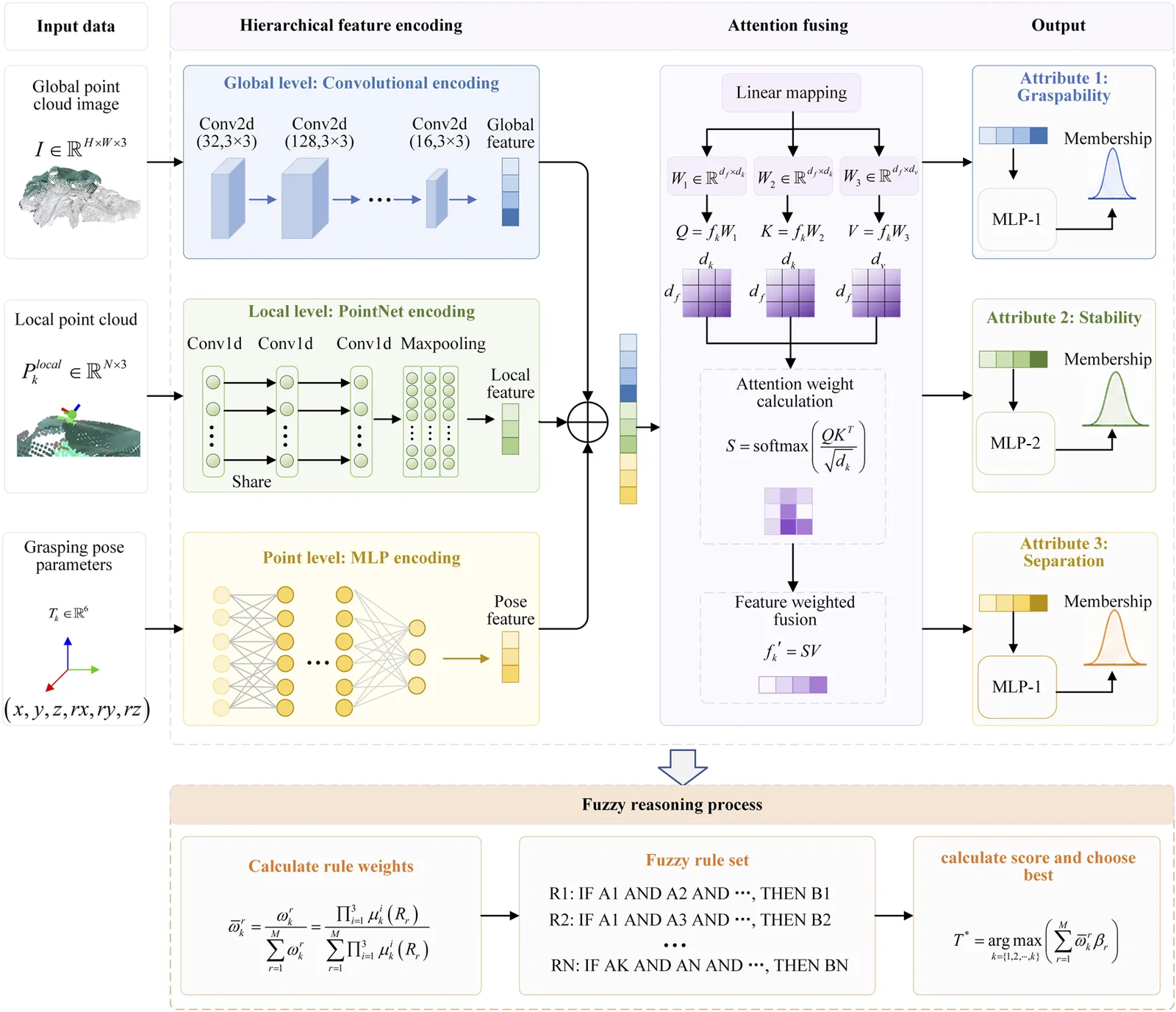
Optimal Grasping Pose Selection Based on the Hierarchical Fuzzy Neural Network. First, a convolutional network, PointNet, and an MLP are used to encode the global point cloud image, the local point cloud corresponding to the candidate pose, and the pose parameters, respectively. Second, multi-level features are adaptively fused through an attention mechanism, and the membership degrees of grasp feasibility, grasp reliability, and grasp separation effectiveness are predicted. Finally, comprehensive reasoning and scoring are performed based on fuzzy rules, and the candidate grasp pose with the highest comprehensive score is selected as the optimal grasp pose.

The inputs of the HFNN are the global point cloud image I , the candidate pose $T_{k}$ , and the corresponding local point cloud $P_{k}^{local}$ . To extract multi-level features, a hierarchical feature extraction module is designed, which consists of a global layer, a local layer, a pose layer, and an attention fusion layer. The global layer takes a fixed-resolution point cloud image as input and uses a convolutional neural network for encoding to extract the overall scene structural feature $f_{k}^{global}$ ; The local layer takes the local point cloud corresponding to the candidate pose as input and uses a PointNet structure to extract the local geometric feature $f_{k}^{local}$ . The pose layer takes the six-dimensional candidate-pose parameter $\left(x,y,z,rx,ry,rz\right)$ as input and maps it using a multilayer perceptron to obtain $f_{k}^{pose}$ . The three branches characterize the overall scene structure, the local morphology of the candidate region, and the grasp-operation parameters, respectively, thereby forming a hierarchical feature representation for candidate-pose attribute evaluation.

To effectively fuse multi-level features, an attention aggregation strategy is further proposed. This strategy takes multi-level features as input and outputs the aggregated attribute feature. Specifically, the three types of features are concatenated to obtain the multi-level input feature $f_{k}$ , as shown in Equation 17.

$$f_{k}=\left[f_{k}^{global},f_{k}^{local},f_{k}^{pose}\right]$$ (17)

To aggregate the multi-level features, three learnable parameter matrices ( $W_{1},W_{2},W_{3}$ ) are used to map $f_{k}$ into different feature spaces, as expressed in Equation 18.

$$\left\{\begin{array}{l} Q=f_{k}W_{1}\\ K=f_{k}W_{2}\\ V=f_{k}W_{3} \end{array}\right.$$ (18)

To measure the correlations among different features, a dot-product operation is performed between the query vector Q and the key vector K , followed by softmax normalization to obtain the feature-importance weight matrix. This weight matrix reflects the contribution of different feature dimensions to attribute modeling. Based on this weight matrix, the value vector V is weighted and aggregated, yielding the fused attribute feature representation, as shown in Equation 19.

$${f_{k}}^{\prime }=\text{softmax}\left(\left(f_{k}W_{1}\right){\left(f_{k}W_{2}\right)}^{T}\right)\left(f_{k}W_{3}\right)$$ (19)

Through the above process, adaptive fusion of multi-level features is achieved, allowing features more strongly related to attribute discrimination to receive higher weights. After obtaining the aggregated attribute feature ${f_{k}}^{\prime }$ , the network adopts a multi-head structure with shared features and independent outputs for membership prediction. Specifically, for the three attributes, namely, grasp feasibility $F_{k}^{1}$ , single-layer separation capability $F_{k}^{2}$ , and grasp stability $F_{k}^{3}$ , one fully connected output head is set for each attribute. Each output head outputs the logits corresponding to the three linguistic variables Low, Medium, and High for the associated attribute, as expressed in Equation 20.

$$\mu_{k}^{i}=\text{Softmax}\left(W_{k}{f_{k}}^{\prime }+b_{k}\right),i=1,2,3$$ (20)

where $\mu_{k}^{i}\in R^{3}$ denotes the membership distribution of the candidate pose over the linguistic variables for the k -th attribute, and $W_{k}$ and $b_{k}$ denote the coefficients of the fully connected layer. After the membership degrees are obtained, the comprehensive score of the candidate pose is calculated through the fuzzy inference process described in Equations 15 and 16, and the optimal grasp pose is determined accordingly.

#### 3.3.3 Loss function design

To effectively train the hierarchical fuzzy neural network, this paper adopts a multi-attribute supervised learning strategy to constrain the membership prediction results of the three attributes. For each candidate pose, annotators assign class labels for three attributes: grasp feasibility, single-layer separation capability, and grasp stability. The labels take values of 0, 1, and 2, corresponding to the three linguistic variables Low, Medium, and High, as defined in Equation 21.

$$y_{k}=\left[y_{k}^{1},y_{k}^{2},y_{k}^{3}\right],y_{k}^{i}\epsilon \left\{0,1,2\right\},i=1,2,3$$ (21)

To avoid unreliable training results caused by class imbalance, samples are screened during data collection and annotation to ensure a relatively balanced distribution of the Low, Medium, and High categories within each attribute. For each attribute, the cross-entropy loss function is used to measure the difference between the predicted membership degrees and the annotated labels, and the overall loss function is defined as the sum of the losses over all attributes. The loss function is defined in Equation 22 as,

$$L=-\sum_{k=1}^{M}\sum_{i=1}^{3}y_{k}^{i}\log\mu_{k}^{i}$$ (22)

During training, the model parameters are updated through backpropagation and the Adam optimizer. The model is supervised only by manually annotated labels for the membership prediction of the three attributes. Fuzzy rule-based reasoning and comprehensive scoring are used for pose evaluation and ranking only during inference. The rule-based scores are preset by experts and are not treated as learnable network parameters; thus, they do not participate in gradient updates. This design ensures consistency between the training objective and the format of the manually annotated data.

#### 3.3.4 Fuzzy rules and score settings

This paper includes three evaluation attributes, and each attribute is described using the three linguistic variables, i.e., Low, Medium, and High. Therefore, the complete rule base contains only 27 rules. Low, Medium, and High are encoded as 0, 1, and 2, respectively. Furthermore, for the i -th candidate pose, the comprehensive attribute level is defined in Equation 23 as,

$$q_{i}=q_{i}^{1}+q_{i}^{2}+q_{i}^{3}$$ (23)

Where $q_{i}^{1},q_{i}^{2},q_{i}^{3}\in \left\{0,1,2\right\}$ denote the encoded values of grasp feasibility, grasp reliability, and grasp separation effectiveness for the i -th candidate pose, respectively.

The fuzzy rules and their scores are defined as follows: when all three attributes are High (i.e., $q_{i}=6$ ), the comprehensive evaluation is Excellent, and the score is set to 100; when two attributes are High and the remaining attribute is Medium (i.e., $q_{i}=5$ ), the comprehensive evaluation is Good, and the score is set to 90; when two attributes are High and the remaining attribute is Low, or when one attribute is High and the other two attributes are Medium (i.e., $q_{i}=4$ ), the comprehensive evaluation is Relatively Good and the score is set to 80; when all three attributes are Medium, or when the three attributes are High, Medium, and Low (i.e., $q_{i}=3$ ), respectively, the comprehensive evaluation is Medium, and the score is set to 70; when one attribute is High and the other two attributes are Low, or when two attributes are Medium and the remaining attribute is Low (i.e., $q_{i}=2$ ), the comprehensive evaluation is Relatively Poor, and the score is set to 60; when one attribute is Medium and the other two attributes are Low (i.e., $q_{i}=1$ ), the comprehensive evaluation is Poor, and the score is set to 50; when all three attributes are Low (i.e., $q_{i}=0$ ), the comprehensive evaluation is Very Poor, and the score is set to 40.

The above setting principles were jointly discussed and agreed upon by several experts with relevant experience. Therefore, they can reflect the consensus decision logic in stacked-fabric grasping tasks and exhibit certain robustness and generalization capability. In summary, the fuzzy rules and their scores are shown in Table 1.

**TABLE 1:** Fuzzy rules and scores.

Rule number	Grasp feasibility	Grasp separation effectiveness	Grasp reliability	$q_{i}$	Overall evaluation	Score
1	High	High	High	6	Very good	100
2	High	High	Medium	5	Good	90
3	High	Medium	High	5	Good	90
4	Medium	High	High	5	Good	90
5	High	High	Low	4	Relatively good	80
6	High	Medium	Medium	4	Relatively good	80
7	High	Low	High	4	Relatively good	80
8	Medium	High	Medium	4	Relatively good	80
9	Medium	Medium	High	4	Relatively good	80
10	Low	High	High	4	Relatively good	80
11	High	Medium	Low	3	Medium	70
12	High	Low	Medium	3	Medium	70
13	Medium	High	Low	3	Medium	70
14	Medium	Medium	Medium	3	Medium	70
15	Medium	Low	High	3	Medium	70
16	Low	High	Medium	3	Medium	70
17	Low	Medium	High	3	Medium	70
18	High	Low	Low	2	Relatively poor	60
19	Medium	Medium	Low	2	Relatively poor	60
20	Medium	Low	Medium	2	Relatively poor	60
21	Low	High	Low	2	Relatively poor	60
22	Low	Medium	Medium	2	Relatively poor	60
23	Low	Low	High	2	Relatively poor	60
24	Medium	Low	Low	1	Poor	50
25	Low	Medium	Low	1	Poor	50
26	Low	Low	Medium	1	Poor	50
27	Low	Low	Low	0	Very poor	40

## 4 Experiments and discussion

### 4.1 Dataset construction

To verify the effectiveness of the proposed method in the stacked-fabric grasp-pose selection task, this paper constructs a dataset for candidate grasp-pose attribute evaluation. The data acquisition platform uses an Intel RealSense D435i camera to acquire RGB images and three-dimensional point clouds of fabric scenes. The experimental objects are stacked fabrics with various morphologies. Complex grasping scenes are constructed through random stacking, entanglement, and local occlusion to simulate practical sorting challenges, such as irregular fabric morphology, complex inter-layer relationships, and unclear operable regions.

For each acquired scene, the corresponding RGB image and raw global point cloud are first saved. The global point cloud is used for convex structure perception and candidate grasp-pose generation. For each candidate grasp pose, a data sample is constructed with three inputs: the global point cloud I , the candidate pose $T_{k}$ , and the corresponding local point cloud $P_{k}^{local}$ . The attributes of each candidate pose are labeled by experts. Specifically, experts evaluate each candidate grasp pose from three dimensions: grasp feasibility, single-layer separation capability, and grasp stability. Each attribute is described using three linguistic variables, namely, Low, Medium, and High, which are encoded as 0, 1, and 2, respectively.

### 4.2 Evaluation metrics

To comprehensively evaluate the effectiveness of the proposed method in candidate grasp-pose evaluation, attribute classification accuracy is used to assess the model’s prediction performance for different grasp attributes. For the k -th attribute, its classification accuracy is defined in Equation 24 as,

$$Acc_{k}=\frac{N_{k}^{correct}}{N},k=1,2,3$$ (24)

where N denotes the total number of candidate pose samples in the test set, and $N_{k}^{correct}$ denotes the number of samples for which the predicted class of the k -th attribute is consistent with the manually annotated class. In this paper, k=1,2,3 correspond to grasp feasibility, grasp separation effectiveness, and grasp reliability, respectively. Furthermore, the mean attribute accuracy is used to measure the overall attribute prediction performance of the model, which is defined in Equation 25 as,

$$Acc_{mean}=\frac{1}{3}\sum_{k=1}^{3}Acc_{k}$$ (25)

### 4.3 Experimental verification and analysis

#### 4.3.1 Model convergence analysis

To verify the training stability of the proposed hierarchical fuzzy neural network, this paper records the changes in the training and validation losses with respect to the number of epochs during training, as shown in Figure 6.

**FIGURE 6:**
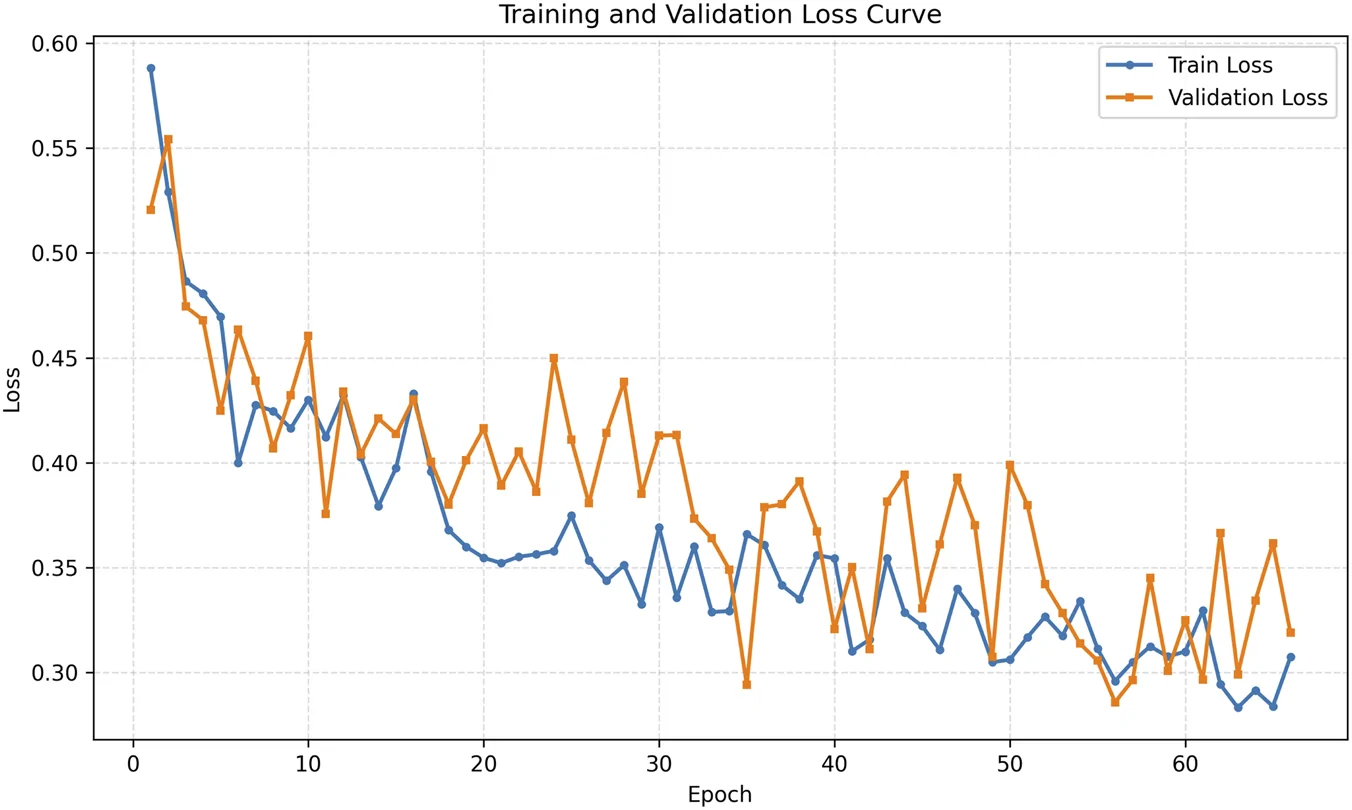
Loss curve.

It can be observed that as the number of training epochs increases, the training loss generally decreases and gradually stabilizes. The validation loss decreases rapidly in the initial stage and then enters a stable convergence state, indicating that the model can effectively learn the mapping relationship between candidate grasp poses and manually annotated attributes. Meanwhile, no obvious gap continuously widens between the training and validation losses, suggesting that the model has good generalization ability on the constructed dataset and does not suffer from severe overfitting. These results indicate that the proposed network structure and training strategy enable stable convergence, providing a reliable basis for subsequent candidate-pose attribute prediction and comprehensive scoring.

#### 4.3.2 Visualization analysis of optimal grasping poses

To further provide an intuitive evaluation of the ability of the proposed method to select optimal poses from candidate poses in complex stacked-fabric scenes, several typical scenes are selected for visualization analysis, as shown in Figure 7. In the figure, the operable convex regions extracted by the convex structure perception method are highlighted, and both the candidate grasp poses and the finally selected optimal grasp pose are overlaid on the corresponding scenes.

**FIGURE 7:**
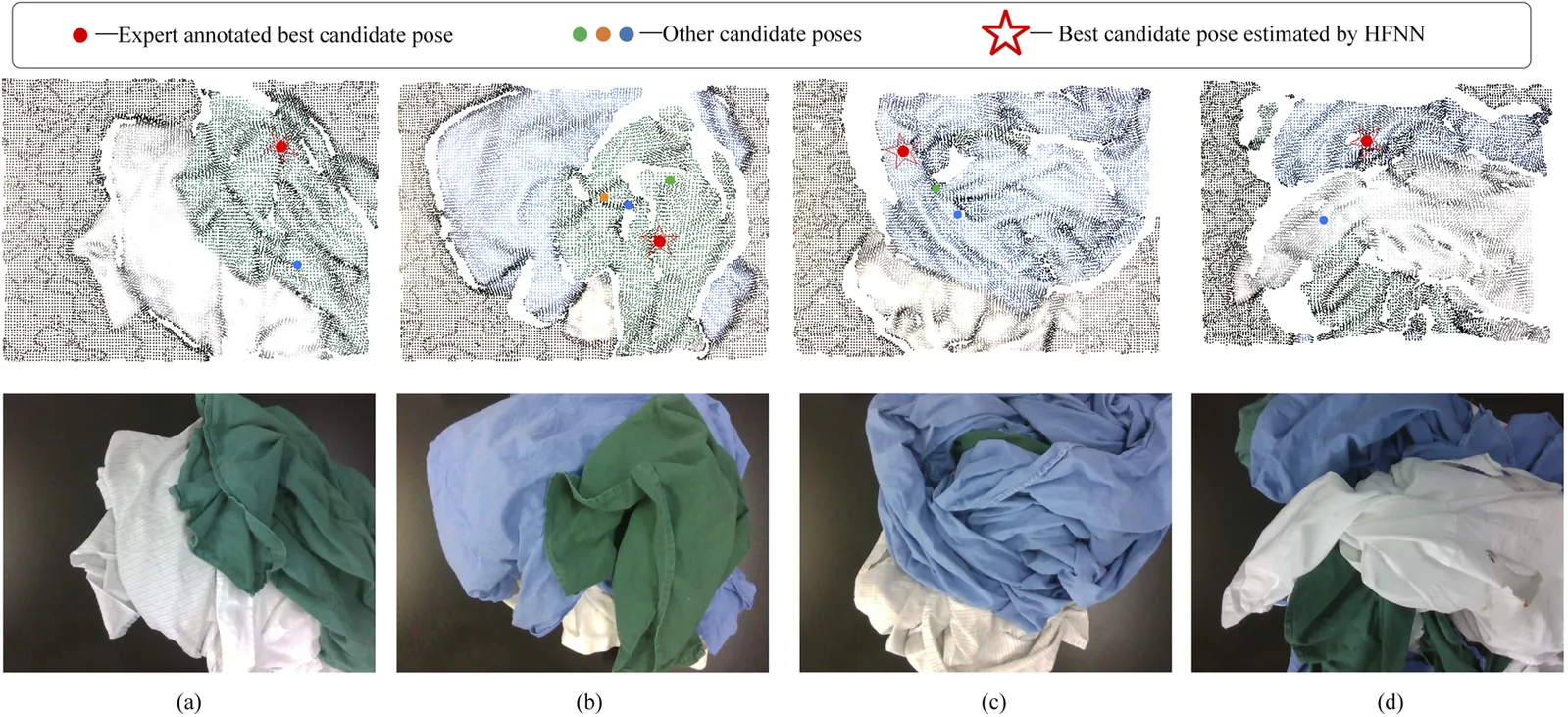
Estimation Results of the Best Grasp Poses in Different Fabric Scenes. **(a)** Two fabric types, two-layer stacking, partial occlusion; **(b)** three fabric types, three-layer stacking, partial occlusion; **(c)** three fabric types, three-layer stacking, entangled occlusion; **(d)** four fabric types, four-layer stacking, entangled occlusion. The top row shows the candidate grasp-pose localization results in the point cloud, and the bottom row shows the corresponding RGB images. The red solid dots denote the optimal candidate poses annotated by experts, the other colored solid dots denote the remaining candidate poses, and the red stars denote the optimal grasp poses predicted by the HFNN.

It can be observed that the proposed method can stably extract convex structural regions on fabric surfaces under different stacking, entanglement, and local occlusion conditions, and localize the optimal pose on structures with clear operational significance. Furthermore, the visualization provides information on the position, orientation, and comprehensive score of the optimal pose, indicating that the hierarchical fuzzy neural network can jointly consider global scene distribution, local geometric morphology, and pose parameters to effectively distinguish and rank candidate poses. The visualization results show that the proposed method can not only generate structurally reasonable candidate poses but also select the optimal grasp pose from the candidate set in a manner that better conforms to expert evaluation patterns.

### 4.4 Comparative experiments and analysis

#### 4.4.1 Comparative methods

To verify the effectiveness of the proposed hierarchical fuzzy neural network in the candidate grasp-pose evaluation task, the proposed method is compared with several baseline models. All learning-based models are trained on the same training set and evaluated on the same test set. To ensure a fair comparison, all models use the same data partitioning, training strategy, and evaluation metrics. The comparison models are as follows:

MLP-Only: This model uses only candidate grasp-pose parameters as input, adopts a multilayer perceptron for feature encoding, and outputs the classification results of the three attributes.

Global-CNN: This model takes the global point cloud as input, uses a convolutional neural network to extract the overall stacked-structure features of the fabric, and predicts the three attributes of candidate poses based on global scene features.

Local-PointNet: This model takes the local point cloud corresponding to the candidate pose as input, uses a PointNet encoder to extract local geometric features, and performs classification prediction for grasp feasibility, grasp separation effectiveness, and grasp reliability.

Direct-Fusion: This model simultaneously takes the global point cloud image, the local point cloud, and the candidate-pose parameters as inputs. A convolutional encoder, a PointNet encoder, and an MLP encoder are used to extract features from these inputs, respectively. The three types of features are then directly concatenated and fed into the classification head. This model is used to verify the role of multi-level information fusion in grasp-attribute prediction and also serves as a comparison model without an attention fusion mechanism.

HFNN: This model is the method proposed in this paper. It fuses global scene information, local geometric features, and candidate-pose parameters, achieves adaptive fusion of multi-level features through an attention mechanism, and learns the membership distributions of three attributes: grasp feasibility, grasp separation effectiveness, and grasp reliability. Subsequently, fuzzy-rule reasoning is combined to obtain the comprehensive score of each candidate pose, which is used to select the optimal grasp pose.

#### 4.4.2 Comparative experiments and analysis

The comparison results of different methods on the test set are shown in Table 2. Bold values indicate the best results for each metric.

**TABLE 2:** Comparative experimental results.

Method	Grasp feasibility prediction accuracy	Grasp separation effectiveness prediction accuracy	Grasp reliability prediction accuracy	Mean accuracy
MLP-only	90.0%	77.0%	90.0%	85.7%
Global-CNN	90.0%	76.7%	90.3%	85.7%
Local-PointNet	91.7%	**82.0%**	91.7%	88.5%
Direct-fusion	96.7%	80.0%	96.0%	90.9%
HFNN	**98.0%**	80.3%	**96.7%**	**91.7%**

Bold values indicate the best results for each metric.

It can be seen that the overall performance of single-input models is lower than that of multi-input fusion models. Among them, MLP-Only relies only on pose parameters and lacks the ability to perceive the structural state of fabrics, resulting in limited attribute prediction performance. Local-PointNet can characterize the local geometric morphology of candidate regions and outperforms MLP-Only in grasp feasibility and grasp stability prediction. However, due to the absence of global scene context, it remains insufficient in evaluating single-layer separation capability. Global-CNN can represent the overall stacking state of fabrics, but it is difficult for it to precisely describe the local geometric details around specific candidate poses. The Direct-Fusion model achieves better results by fusing global, local, and pose information, indicating that multi-level information plays an important role in candidate-pose evaluation. Compared with the above methods, the proposed HFNN further uses an attention mechanism to adaptively assign the importance of features at different levels and performs comprehensive decision-making over multi-attribute memberships through fuzzy inference, thereby achieving the best attribute prediction accuracy. These results indicate that the proposed method can more effectively learn expert fuzzy evaluation patterns for candidate grasp poses and improve the reliability of comprehensive candidate-pose ranking.

### 4.5 Robotic fabric sorting experiment

To verify the engineering feasibility of the proposed method, a robotic sorting platform is constructed. During the experiments, randomly stacked scenarios are set up to conduct robotic sorting trials. The robotic sorting process is shown in Figure 8.

**FIGURE 8:**
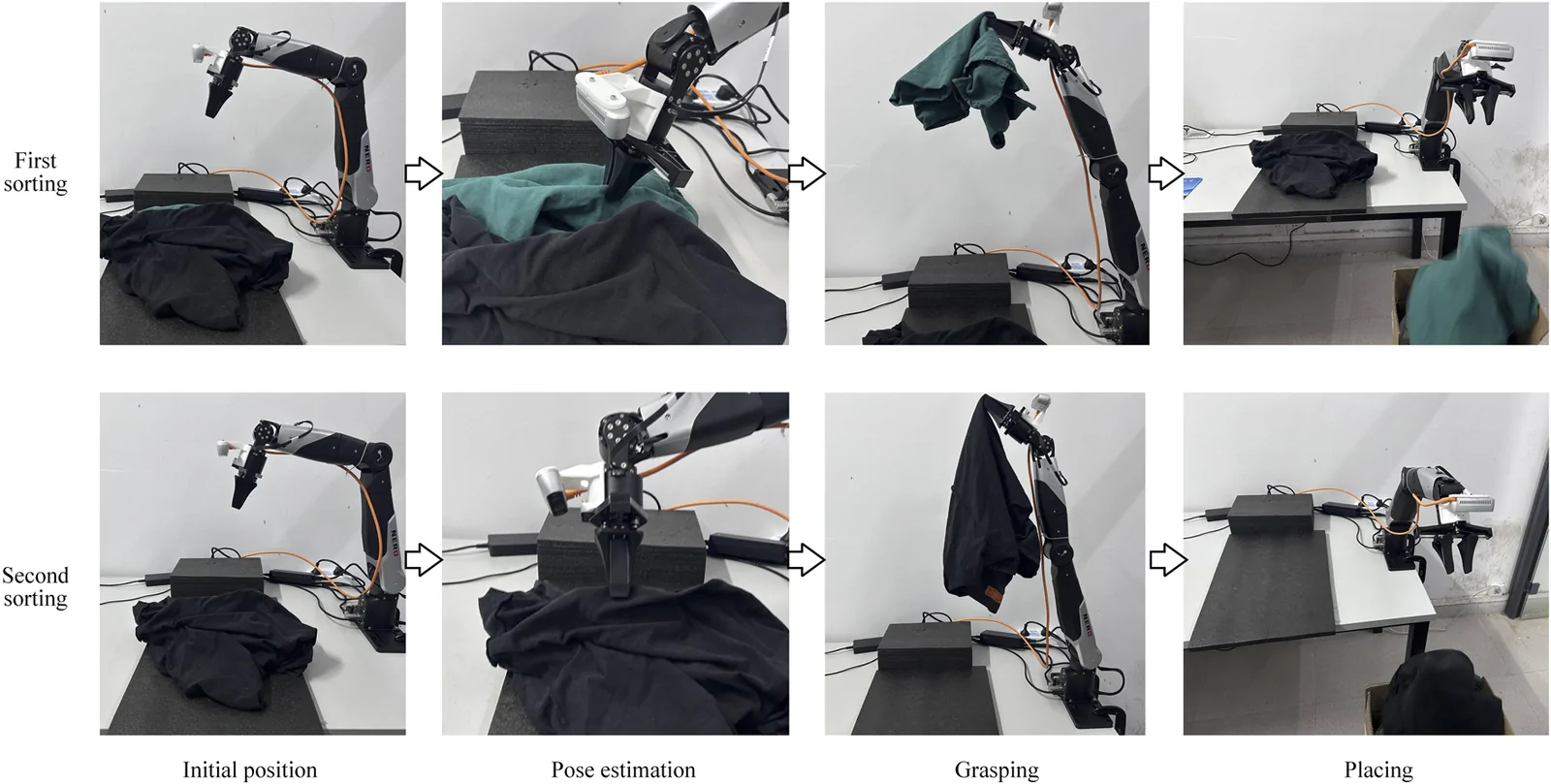
Robotic sorting process (including two sorting operations, each consisting of three stages: pose estimation, grasping, and placement).

Furthermore, to evaluate the effectiveness of the convex structure perception module and the HFNN-based fuzzy inference module, the following two comparison methods are proposed:

Method 1: Candidate grasp poses are generated using the convex structure perception module, and the best grasp pose is selected from the candidate set based on a manually designed scoring method.

Method 2: A set of candidate grasp poses is generated using the convex structure perception module, and the optimal grasp pose is selected using the HFNN-based fuzzy inference module.

A total of 30 robotic sorting experiments are conducted, and the grasp success rate is recorded. The experimental results show that Method one achieved a grasp success rate of 80.0% in randomly stacked scenarios, indicating that the candidate grasp poses extracted by the convex structure perception module can achieve satisfactory sorting performance. Method 2 achieved a grasp success rate of 93.3%. Compared with Method 1, Method 2 improved the grasp success rate by 13.3%, demonstrating that the HFNN-based fuzzy inference module can effectively evaluate candidate poses and select better grasp poses. These results verify the effectiveness of the two core modules for robotic grasping.

## 5 Conclusion

At present, robotic grasping of stacked fabrics remains highly challenging. Because fabrics are flexible, easily deformable, self-occluding, and structurally irregular, robots struggle to stably identify grasp regions with operational significance in complex scenes and to select the optimal grasping strategy from multiple feasible candidate poses. By contrast, human experts can rapidly complete grasp-pose selection by relying on the perception of local convex structures and an experience-driven fuzzy judgment mechanism. Learning and simulating human structural perception and fuzzy decision-making capabilities in fabric grasping is an important approach to improving robotic manipulation of complex deformable objects.

This paper proposes a stacked-fabric grasping method based on convex structure awareness and hierarchical fuzzy decision-making. The complex fabric grasping task is modeled as a staged decision-making process consisting of convex structure-region perception, candidate grasp-pose generation, multi-attribute fuzzy evaluation, and optimal pose selection. Specifically, a fabric convex structure-region perception method based on point cloud geometric features is first proposed. By fusing local curvature, normal consistency, and spatial clustering information, the method extracts convex structural regions with operational significance from complex stacked point clouds and generates a set of candidate grasp poses satisfying both structural constraints and gripper-width constraints. Furthermore, to address the difficulty of precisely evaluating candidate grasp poses, a hierarchical fuzzy neural network model is proposed. The model extracts multi-level features from the global point cloud image, the local point cloud corresponding to the candidate pose, and the six-dimensional pose parameters. It then achieves adaptive feature fusion through an attention mechanism and learns the membership distributions of candidate poses over three attributes. Finally, expert fuzzy rules are combined to perform comprehensive reasoning over multi-attribute memberships, enabling interpretable scoring and optimal selection of candidate grasp poses. Experimental results show that the proposed method can stably extract convex structural regions in complex fabric scenes. The proposed method overall outperforms the comparison methods in terms of grasp feasibility prediction accuracy, grasp separation effectiveness prediction accuracy, grasp reliability, prediction accuracy, and mean accuracy.

## Data Availability

The raw data supporting the conclusions of this article will be made available by the authors, without undue reservation.
